# Influence of house characteristics on mosquito distribution and malaria transmission in the city of Yaoundé, Cameroon

**DOI:** 10.1186/s12936-020-3133-z

**Published:** 2020-01-30

**Authors:** Carmene S. Ngadjeu, Patricia Doumbe-Belisse, Abdou Talipouo, Landre Djamouko-Djonkam, Parfait Awono-Ambene, Sevilor Kekeunou, Wilson Toussile, Charles S. Wondji, Christophe Antonio-Nkondjio

**Affiliations:** 1 Institut de Recherche de Yaoundé (IRY), Organisation de Coordination pour la lutte Contre les Endémies en Afrique Centrale (OCEAC), P.O. Box 288, Yaoundé, Cameroon; 2 Faculty of Sciences, University of Yaoundé I, P.O. Box 337, Yaoundé, Cameroon; 3 Faculty of Sciences, University of Dschang, P.O. Box 337, Dschang, Cameroon; 4 Ecole Nationale Supérieure Polytechnique University of Yaoundé I, P.O. Box 8390, Yaounde, Cameroon; 5 Vector Biology Liverpool School of Tropical Medicine Pembroke Place, Liverpool, L3 5QA UK; 6 Faculty of Medicine Paris-Sud, 63 rue Gabriel Peri, 94276 Le Kremlin-Bicêtre, Paris, France

**Keywords:** Malaria transmission, Houses characteristics, Culicines, Anophelines, *An. gambiae*, Yaoundé

## Abstract

**Background:**

Improving house structure is known to limit contact between humans and mosquitoes and reduce malaria transmission risk. In the present study, the influence of house characteristics on mosquito distribution and malaria transmission risk was assessed in the city of Yaoundé.

**Methods:**

The study was conducted from March 2017 to June 2018 in 32 districts of the city of Yaoundé. Mosquito collections were performed indoor in 10 to 15 houses per district using CDC light traps. A total of 467 houses, selected randomly were used. A pretested questionnaire was submitted to participants of the study to collect information on the household: the number of people per house, education level, type of walls, presence of ceilings and eaves, number of windows, usage of long-lasting insecticidal nets (LLINs), number of bedroom and number of window. Mosquitoes collected were identified morphologically. Anophelines were tested by ELISA to detect infection by *Plasmodium* parasites. General Estimating Equations adjusting for repeated measures in the same house fitting negative binomial analysis were used to assess the influence of house characteristics on mosquito distribution.

**Results:**

A total of 168,039 mosquitoes were collected; *Culex* spp emerged as the predominant species (96.48%), followed by *Anopheles gambiae* sensu lato (*s.l*.) (2.49%). Out of the 1033 *An. gambiae s.l*. identified by PCR, 90.03% were *Anopheles coluzzii* and the remaining were *An. gambiae* sensu stricto (*s.s*.) (9.97%). The high number of people per household, the presence of screens on window and the possession of LLINs were all associated with fewer mosquitoes collected indoors, whilst opened eaves, the high number of windows, the presence of holes in walls and living close to breeding sites were associated with high densities of mosquitoes indoor. Out of 3557 Anophelines tested using ELISA CSP, 80 were found infected by *Plasmodium falciparum* parasites. The proportion of mosquitoes infected did not vary significantly according to house characteristics.

**Conclusion:**

The study indicated that several house characteristics such as, the presence of holes on walls, opened eaves, unscreened window and living close to breeding sites, favored mosquito presence in houses. Promoting frequent use of LLINs and house improvement measures, such as the use of screen on windows, closing eaves, cleaning the nearby environment, should be integrated in strategies to improve malaria control in the city of Yaoundé.

## Background

Malaria remains a major public health threat in Cameroon. It is estimated that over 24% of the 25 million Cameroonian have at least one malaria attack yearly [1]. Disease prevention relies mainly on the use of long-lasting insecticidal nets (LLINs) [2, 3]. Although the massive scale up of these tools between 2000 and 2015 permitted substantial decrease of malaria morbidity and mortality across Cameroon and Africa [1, 4], control measures are threatened by poor coverage and sub-standard nets, the rapid expansion of insecticide resistance, and changes in vector feeding and biting behaviour [3, 5]. In Cameroon, the disease is still largely prevalent in both urban and rural settings [1, 6]. The city of Yaoundé, is also considered as highly affected by the disease [1]. The city has seen it population multiply by 8 in less than 3 decades with intense migration of population from rural to urban settings [7, 8]. The persistence transmission of malaria in Yaoundé is considered to result from the frequent influx of migrants coming from rural settings where malaria is hyperendemic, and from an increase in unplanned urbanization, characterized by the extension of human settlements in wetland and the colonization of swamps for the practice of urban agriculture which favored vector population distribution and maintenance [9–11]. In Cameroon, the number of cities with more than 50,000 inhabitants has increased from 2 in the 1970s to over 50 nowadays [8, 12].

The two main cities of the country Yaoundé and Douala have each around 3 million inhabitants. It is estimated that over 52% of the population now live in urban settings [7, 8]. During the last decade, increase living standards and wealth in urban settings let to housing improvements such as the replacement of traditional houses made of mud with thatched roofs by modern houses constructed with concrete cement blocks with corrugated metal and tiled roofs [13–16]. Modern constructions or well-constructed houses have been reported to provide high protection against mosquito bites and malaria transmission compare to traditional style houses [16]. Yet the rapid demographic growth of cities also saw the fast development of informal settlements [17]. In sub-Saharan Africa cities, it is estimated that 47% of the urban population lives in informal settlements or poorly constructed houses [13, 17]. Up to date the influence of house characteristics on the exposition to vector borne diseases transmission has not been fully addressed. In Yaoundé, well-developed districts are often surrounded by poorly constructed houses or shanty towns [17]. Although there are increasing reports suggesting outdoor transmission of malaria also occurring in various epidemiological settings [5, 11, 18], most malaria transmission cases, still occurs indoors at night [19]. The following indicating that houses if not well isolated, could expose inhabitants to high transmission risk [20]. Studies conducted in East and West Africa indicated that houses with open eaves and those with no ceilings were associated with increased mosquito nuisance and higher level of malaria compare to those with closed eaves and ceilings [15, 21–23] Therefore understanding factors exposing the population to mosquito bites and malaria transmission in houses could help design strategies that could improve people protection when they are at home. Placing screen on windows in houses, closing eaves, and placing ceilings are interventions which have been largely used in many countries to fight against malaria vectors [15, 24]. These additional measures provide protection to the entire household by decreasing contact between mosquitoes and humans [25] and also protecting against nuisance and transmission of other mosquito borne diseases [24, 26]. House improvement have shown to be efficient for controlling malaria transmission and mosquito burden irrespective of the level of transmission in East Africa [15, 27]. Up to date, there is still not enough information on the influence of house characteristics parameters such as the type of building materials, presence or absence of eaves, ceilings and screening over windows, number of inhabitants per households, use of LLINs on the indoor abundance of mosquitoes and malaria transmission pattern in forested or urban settings in Central Africa.

In Cameroon malaria transmission is perennial in most part of the country; and, up to 15 Anophelines species are considered as malaria vectors [28]. In urban settings malaria transmission is vectored by species, such as *Anopheles gambiae*, *Anopheles coluzzii* and *Anopheles funestus* [11, 29]. Although there are more and more studies reporting perennial malaria transmission in urban settings, factors affecting the dynamic of the disease are still not well understood as well as the performance of treated nets in different type of houses.

In the course of the present study, the influence of different house characteristics on the distribution of mosquitoes and malaria transmission risk in the city of Yaoundé Cameroon was assessed.

## Methods

### Study areas

**Fig. 1:**
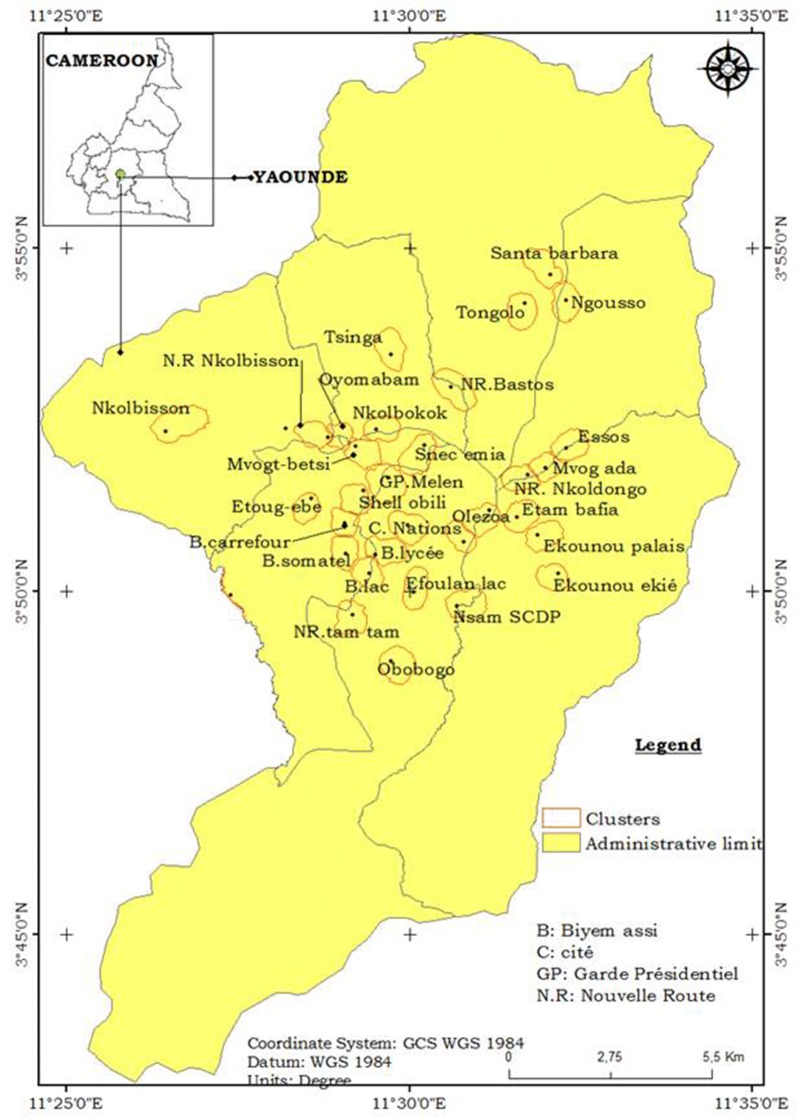
A map of Yaoundé city showing study sites (Source: National Institute of Cartography, Cameroon)

Mosquito collections were conducted in the city of Yaoundé (3°51′N 11°30′E) the capital city of Cameroon. The city is situated within the Congo-Guinean phytogeographic domain, characterize by an equatorial climate with four seasons: two rainy seasons extending from March to June and September to November and two dry seasons extending from December to February and July to August. Yaoundé is the second largest city of the country with about 3 million inhabitants. The city is drained by several permanent streams. The average rainfall in Yaoundé is estimated at 1688 mm/year, the average annual temperature is 26.31 °C varying between 16 and 33 °C depending on the season. The average humidity is 80% and varies during the day between 35 and 98% [30]. The city is exposed to frequent humid winds blowing South-West to West or North to West [31]. Samples were collected in houses in 32 districts (Fig. 1 ). All the districts were characterized by the presence of highland and lowland areas. Several river’s systems are distributed within the city these include river Mfoundi, Biyeme, and Mefou. Most of the districts are highly populated areas with constructions in both highland and lowland areas. Marshlands along rivers are exploited for house construction and the practice of market gardening during the dry season. These areas are the main sources of breeding habitats for mosquitoes. The study sites extended from the city centre to the periphery (Fig. 1).

### Household survey

For field surveys, each district was divided into 10 different sectors including each 20 to 50 houses. Sectors were situated 50 to 100 m apart. One house was randomly selected from each sector for mosquito collection. If consent was not obtained from a selected household a neighbouring household was chosen.

A structured questionnaire was submitted to the heads of households where mosquito collections were performed. A total of 467 households were included in the study. Surveys were regularly conducted in 350 houses (once every 2 months (collection period) during the seven collection periods of the study). In the remaining 117 houses, surveys were conducted during less than 4 collection periods.

The following formula was used for sample size calculation

$${\text{n }} = \, \varepsilon^{ 2} *{\text{p}}*{\text{q}}/{\text{i}}^{ 2}$$

with n = sample size, p the probability to choose a house of “type A” and q the probability of not choosing “type A” house, Ɛ = 1.96, i = 0.05.

All houses surveyed were concurrently sampled for mosquitoes. A pre-tested questionnaire including general information on the demographic and size characteristics of the household (e.g. number of people living in the houses, number of bedroom and window found in the houses), personal information (study level of household heads) malaria prevention measures (e.g. windows protection, possession and use of mosquito net, uses of others protection methods) was prepared. After preparing the questionnaire, internal reviews were undertaken by three researchers to assess clarity of questions and their interpretability. A pilot study was subsequently conducted to test for validity, internal consistency and reliability of the questionnaire.

Interviewers were trained on how to use the questionnaire and on methods to approach respondents and obtain consent. Parents (household heads, their spouses or an elder representative of the house) who consented to participate in the study, were interviewed. Interviews were undertaken in French or English and in private to reduce the influence from others people. In addition, further information was recorded through direct observation: house construction materials (type of walls), presence/absence of eaves, presence/absence ceilings, presence/absence of vegetation and potential breeding sites. The geographic coordinates of each house was recorded using a hand held global positioning system receivers (Garmin eTrex^®^ GPS).

The following variables were recorded from each house selected for the study: the presence/absence of vegetation (grasses covering at least 50 metres square) around the house, presence absence of a permanent or semi-permanent water source, presence/absence of eaves, holes or screens. The number of windows or doors, the availability of LLINs in the house, the number of people living in the house.

### Adult mosquito sampling and processing

Mosquito sampling was undertaken once every 2 months in each district from March 2017 to June 2018, using Center for Disease Control (CDC) light-traps. Collections were undertaken in the same houses; 10 to 15 houses during three consecutive days per district once every 2 months. One CDC-LTs was placed per house. Traps were placed at about 1 m above the ground, next to a bed with a person sleeping under a treated net from 19:00 to 06:00 h.

Mosquitoes collected were separated using morphological identification keys of Edwards [32]. Anophelines were identified to species using the identification keys of Gillies and Coetzee [33] and Gillies and De Meillon [34]. Anophelines species were stored individually in labeled tubes containing desiccant and kept at − 20 °C for further analyses.

### Laboratory processing of Anophelines

Polymerase chain reaction (PCR) was used to distinguish members of the *An. funestus* group using the protocol of Koekemoer et al. [35]. The insertion polymorphisms of SINE200 retrotransposons within speciation islands was used to identify members of the *An. gambiae* complex [36]. DNA extracted from a leg or a wing according to the livak method [37] was used for these analyses. The heads and thoraxes of female Anophelines were tested for the presence of the circumsporozoite proteins (CSP) of *Plasmodium falciparum* by ELISA, as described by Fontenille et al. [38].

### Data analysis

The CSP rate was calculated as the ratio of mosquitoes infected over mosquitoes tested. The entomological inoculation rate (EIR) (the number of infected bites per person per night ib/p/n) for the CDC light traps, was estimated as follows EIR = 1.605 × (no. of sporozoite positive ELISAs/no. of mosquitoes tested) × (no. of mosquitoes collected/no of CDC light traps). The 1.605 representing the factor of overestimation of human landing catches compare to light traps. Data recorded were entered into Microsoft Excel database and they were later cleaned to check for inconsistencies in data entry and responses. The data was analysed using R software version 3.5.1 [35, 36] and the following packages: Rcmdr, dplyr, ggplot2, knitr, fitdistrplus, gmodels, ggfortify, MASS and tidyr were used. Mean, variance, standard deviation and frequencies were calculated on quantitative data, and frequencies on categorical data. All statistical tests were performed at the significance level of 5%. Proportions were compared using Chi squared test. Since the normality test was significant on the mosquitoes count, we used the Kruskal–Wallis test to assess significant differences of mosquito abundance according to the month and the year. For each house characteristic, the mean mosquito ratio was calculated to compare the different modalities to a reference. Odds Ratios (OR) and the Relative Risk (RR) and their 95% Confidence Intervals (95% CI) were calculated, to assess correlation between house characteristics and mosquito distribution. The number of people per house as well as the number of doors, bedrooms and windows were analysed as both continuous and categorical variables. Multivariate analysis with mosquito count as outcome and houses characteristics as explanatory variables were conducted using mixed effects regression models, to take into account repeated mosquito collections in the same house. The Poisson negative binomial models and the zero inflated variants relative quality were assessed using the Akaike Information Criterion (AIC). The negative binomial model, was used to select variables significantly associated with mosquito distribution according to house characteristics using a backward step-wise procedure based on AIC. Rows with more than one missing variable were not considered during calculations.

## Results

### Indoor mosquito collections

A total of 168,039 (21.64 mosquitoes/trap/night) mosquitoes were collected using 7515 CDC LTs-night. *Culex* spp. was the most abundant representing 96.48% of the total mosquitoes collected, followed by *Anopheles* spp. (2.49%), *Mansonia* spp. (0.47%) and *Aedes* spp. (0.13%) (Table 1). Culicines species recorded included (*Culex quinquefasciatus, Cx duttoni, Cx perfuscus, Aedes albopictus, Ae. aegypti, Mansonia uniformis* and *Coquilletidia sp*). Amongst Anophelines, *Anopheles gambiae* sensu lato (*s.l*.) (0.56 mosquitoes/trap/night) was the most prevalent species followed by *Anopheles funestus s.l.* (0.07 mosquitoes/trap/night) and *Anopheles ziemanni*. Out of the 1033 *An. gambiae s.l.* analysed, 90.03% (n = 930) were *Anopheles coluzzii* and the remaining were *An. gambiae* sensu stricto (*s.s*.) (9.97%). Amongst the 112 *An. funestus s.l.* tested, 91.93% (n = 103) were *An. funestus s.s.* and 8.04% (n = 09) *Anopheles leesoni.*

**Table 1:** Abundance of mosquitoes trapped indoor using CDC light trap in Yaoundé from March 2017 to June 2018

Species	N	Mean/trap	%
*An. funestus s.l.*	540	0.07	0.32
*An. gambiae s.l.*	4181	0.56	2.49
*An. ziemanni*	177	0.02	0.11
Total anophelines	4898	0.65	2.91
*Aedes* spp.	221	0.03	0.13
*Coquillettidia* spp.	8	0.001	0.005
*Culex* spp.	162,129	21.57	96.48
*Mansonia* spp.	783	0.10	0.47
Total culicines	163,141	21.70	97.09
Overall	168,039	22.35	100.00

*N* number

### Monthly variation of mean number of mosquito

**Fig. 2:**
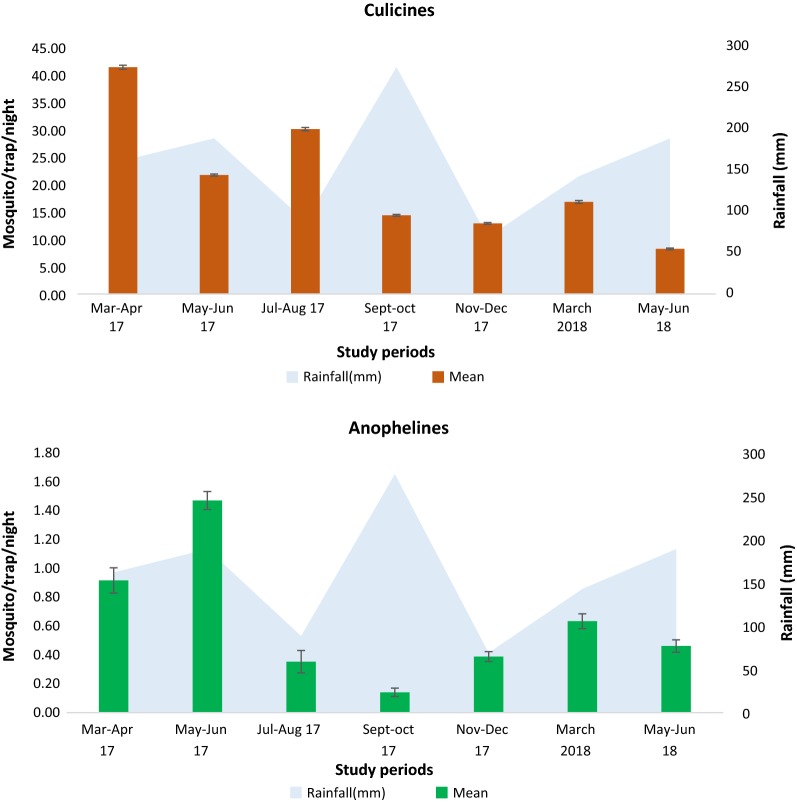
Monthly variation of the average number of Culicines and Anophelines collected per house per night using CDC light traps (error bars represent 95% confidence interval) (Mean: mosquito/trap/night)

The mean number of mosquito was found to vary significantly according to the collection period (Kruskal–Wallis X^2^ = 323.47, p < 2.2e^−16^). In general, high mosquito densities (mean = 42.22 ± 0.36) were recorded at the onset of the short raining season (March/April 2017) before decreasing significantly (8.69 ± 0.20) at the end of the next short raining season (May/Jun 2018). Anophelines densities reached theirs peaks in May/June 2017 (1.47 ± 0.07), before declining in September/October 2017 (0.14 ± 0.02) (p < 0.05). Culicines species were found to be significantly more abundant in March/April 2017 (41.30 ± 0.36) compare to May/June 2018 (8.23 ± 0.19) (Kruskal–Wallis X^2^ = 163.7, p < 2.2e^−16^) (Fig. 2).

### Description of households’ characteristics

A total of 467 houses were surveyed. Most household heads had the secondary school education level (56.01%). More than half of the houses (56.04%) were constructed with cement. Most houses had open eaves (60.49%) and no ceiling under the roof (62.85%), whereas 68.21% had no hole on the walls. The average number of people per house varied between 4 and 6 persons/household. Most houses had less than 5 windows/house (69.19%) and less than 5 bedrooms/house (91.11%). In most houses 87.02% of beds had LLINs. Over 89% of the households indicated using LLINs regularly (Table 2).

**Table 2:** Bivariate analysis comparing house characteristics and mosquito distribution in Yaoundé

Characteristics	No of night collection/house^a^	% of houses	Mos/house/night (CI)	RR (95%CI)	P value
Wall type					
Cement	28,419	56.04	3.01 (2.99–3.03)	0.94 (0.87–1.01)	0.09
Wood	11,667	23.00	4.27 (4.23–4.31)	1.03 (0.95–1.12)	0.52
Mud	3949	7.79	2.18 (2.14–2.23)	1	1
Mix	6680	13.17	2.78 (2.74–2.82)	0.95 (0.86–1.04)	0.23
Occupants					
(1–3)	825	10.87	24.98 (24.64–25.32)	1	–
(4–6)	3296	43.36	20.20 (20.05–20.35)	0.94 (0.87–1.01)	0.07
(7–10)	2728	35.88	20.20 (20.04–20.37)	0.97 (0.91–1.01)	0.43
≥ 11	759	9.89	18.15 (17.84–18.45)	0.93 (0.85–1.02)	0.12
Hole on wall					
Yes	15,415	31.79	4.02 (3.99–4.05)	1.05 (1.00–1.10)	0.03
No	33,082	68.21	2.71 (2.70–2.23)	1	–
Eaves status					
Closed	19,273	39.51	2.63 (2.61–2.65)	1	–
Opened	29,807	60.49	3.62 (3.60–3.64)	1.06 (1.01–1.11)	0.009
Ceiling status					
Present	20,150	37.15	2.58 (2.61–2.61)	1	–
Absent	33,841	62.85	3.17 (3.15–3.19)	1.13 (1.08–1.18)	< 0.0001
Window					
Screened	7834	15.98	3.03 (2.99–3.05)	1	–
Unscreened	41,179	84.02	3.18 (3.16–3.19)	1.09 (1.02–1.15)	0.007
Possession of LLINs					
Yes	39,596	87.02	3.39 (3.37–3.41)	1	–
No	5799	12.98	3.99 (3.93–4.04)	0.92 (0.87–0.98)	0.007
Use of LLINs					
Yes	47,205	89.19	3.02 (3.01–3.05)	1	–
No	5523	10.81	2.51 (2.46–2.55)	0.99 (0.93–1.06)	0.85
Vegetation					
Yes	40,195	79.19	3.35 (3.33–3.37)	1.09(1.03–1.14)	0.002
No	10,562	20.81	2.60 (2.57–2.63)	1	–
Breeding sites					
Yes	41,887	82.39	3.31 (3.29–3.33)	1.05(0.99–1.11)	0.008
No	8954	17.61	2.67 (2.64–2.71)	1	–
Household heads’education level					
Illiterate	1909	4.77	2.69 (2.62–2.77)	1	–
Primary	9926	24.80	3.42 (3.38–3.6)	1.04 (0.93–1.16)	0.53
Secondary	22,418	56.01	3.49 (3.47–3.51)	1.00 (0.89–1.11)	0.99
University	5772	14.42	3.06 (3.02–3.11)	0.95 (0.84–1.07)	0.4
Bedroom					
< 5	40,694	91.11	3.12 (3.10–3.14)	1	–
≥ 5	3971	8.89	3.04 (3.02–3.06)	0.95 (0.88–1.03)	0.22
Window					
< 5	30,021	69.19	3.04 (3.03–3.07)	1	–
≥ 5	13,369	30.81	3.37 (3.34–3.40)	0.99 (0.95–1.04)	0.66

^a^No of night collection/house: represents the total number of collections per night in each house during the course of the study

Compared to houses constructed with mud, houses constructed with wood recorded high densities of mosquitoes (RR = 1.03, 95% CIs 0.95–1.12; P = 0.52) < 0.00). The following characteristics of houses were found to be associated with high mosquito densities: presence of holes on walls (RR = 1.05, 95% CIs 1.00–1.10; P = 0.03), presence of open eaves (RR = 1.06, 95% CIs 1.01–1.11; P = 0.009), absence of ceiling (RR = 1.13, 95% CIs 1.08–1.18; P < 0.0001), unscreened windows (RR = 1.09, 95% CIs 1.02–1.15; P = 0.007), presence of vegetation (RR = 1.09, 95% CIs 1.03–1.14; P = 0.002) and breeding sites close to the house (RR = 1.05, 95% CIs 0.99–1.11; P < 0.008). The number of inhabitants/house, window and bedroom, usage of LLINs and household heads’education were not found to influence mosquito distribution (Table 2).

### Relationship between house characteristics and entomological indicators

**Fig. 3:**
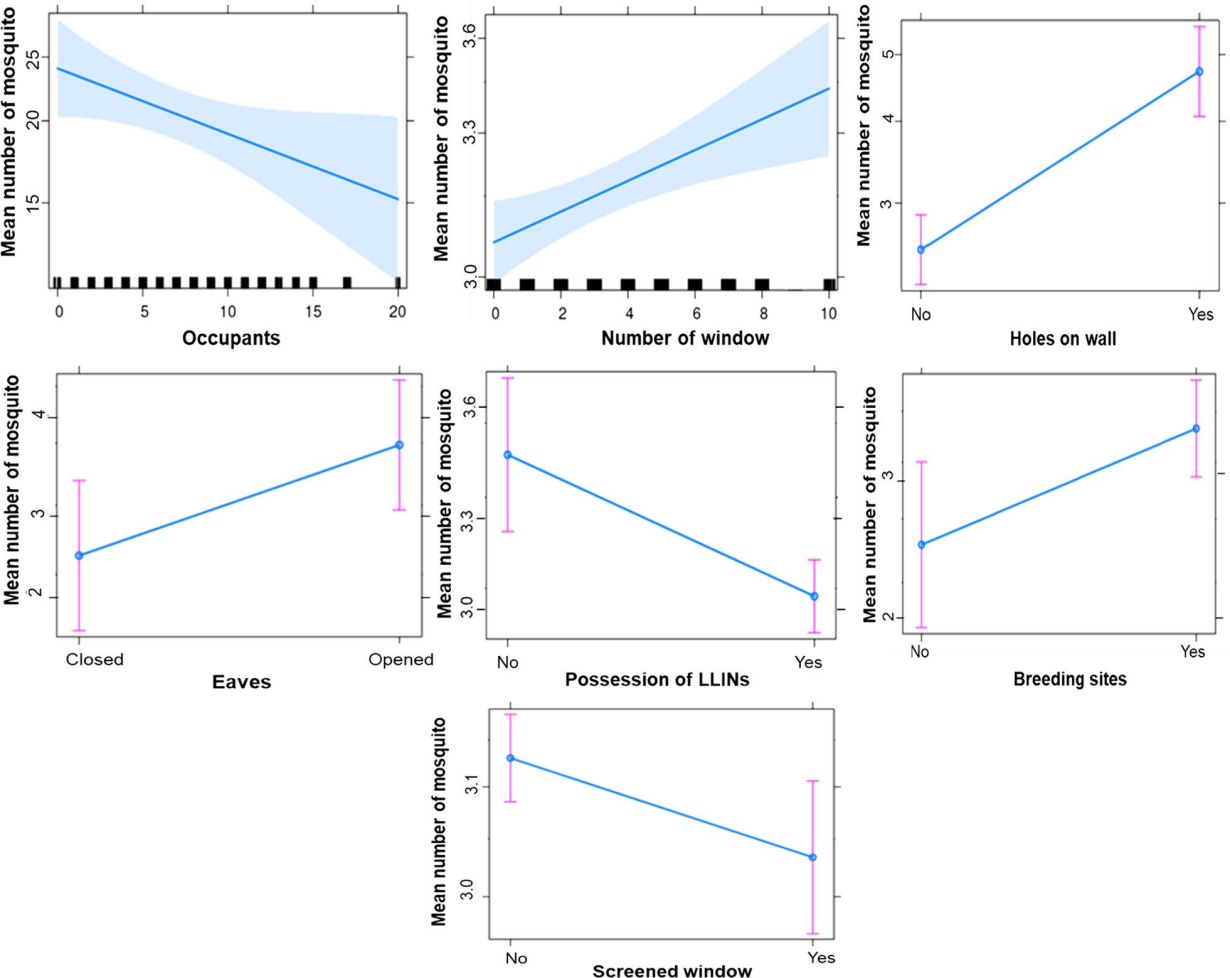
Graphics of the gamma linear regression analysis showing significant variations of the average number of mosquitoes collected according to occupants, number of window, holes on the wall, eaves, use of LLINs breeding sites presence and screens on windows

*House characteristics and mosquito abundance* When all house characteristics were included in a multivariate analysis to assess those strongly influencing mosquito distribution, 04 characteristics including opened eaves (R = 0.17; P = 0.009), the number of window (R = 0.05; P = 0.002), presence of breeding sites (R = 0.21; P = 0.006), holes on the walls (R = 0.49; P < 0.0001) were associated with increased mosquitoes densities in houses. The high number of people per house (R = − 0.2, P = 0.03), the presence of screens on windows (R = − 0.2; P = 0.009) and ownership rate of LLINs (R = − 0.35; P = 0.0004) were associated with less mosquitoes inside houses (Fig. 3).

**Fig. 4:**
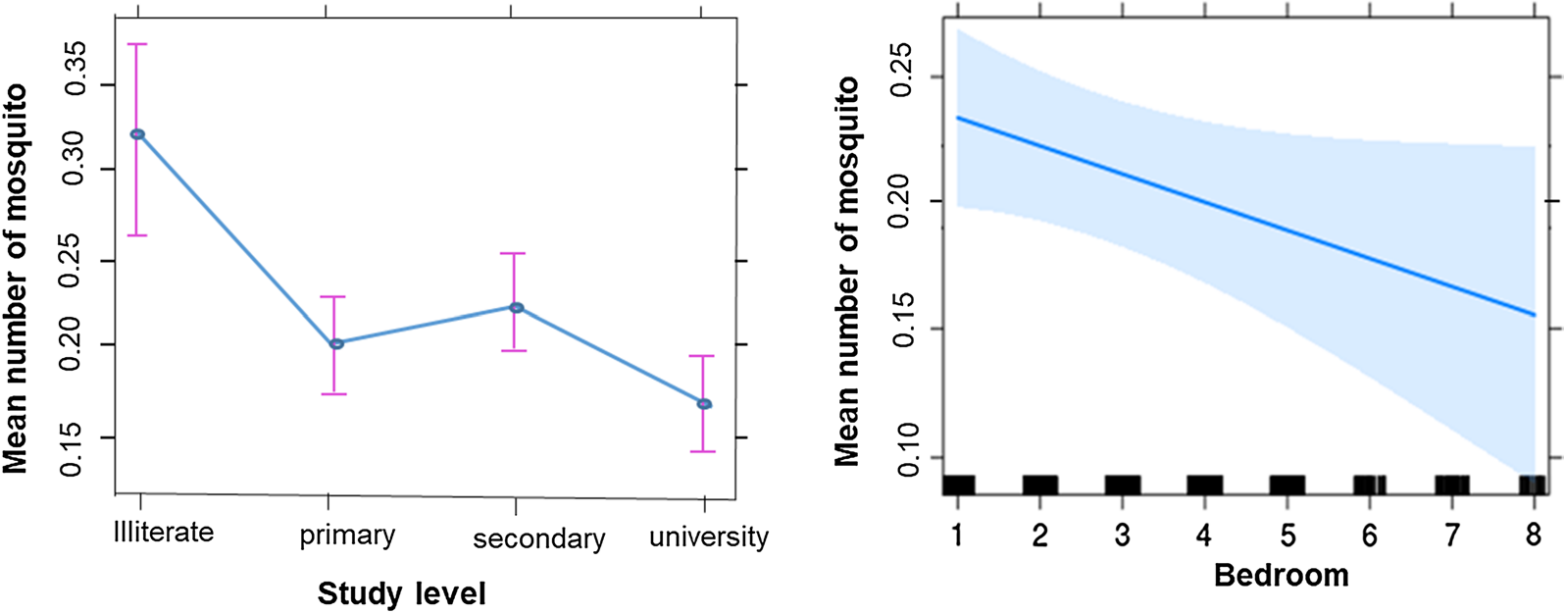
Graphics of the gamma linear regression analysis showing significant variation of the average number of Anophelines collected according to the household heads’ study level and number of bedroom

*House characteristics and Anophelines mosquito’s abundance* The density of Anophelines collected in mud houses was higher than in cement houses (P = 0.02) (Table 3). Bivariate analysis indicated that A high Anophelines densities in houses was closely associated with the presence of open eaves (RR = 1.12, 95% CIs 1.02–1.24; P = 0.014), absence of ceiling (RR = 1.37, 95% CIs 1.24–1.52; P < 0.0001), presence of vegetation (RR = 1.07, 95% CIs 0.01–1.13; P = 0.02) and the presence of vegetation (RR = 1.14, 95% CIs 1.01–1.28; P = 0.03). When multivariate regression analysis were conducted, only 2 parameters remained significant (Fig. 4). These included: number of bedrooms per house and study level of the head of the household.

**Table 3:** Mean number of Anophelines and Culicines collected according to house characteristics in Yaoundé

Features	Anophelines			Culicines		
	Mean (CI)	RR (95% CI)	P value	Mean (CI)	RR (95% CI)	P value
Wall type						
Cement	0.19 (0.18–0.19)	0.8 (0.67–0.96)	0.02	5.12 (5.09–5.16)	0.99 (0.91–1.08)	0.77
Wood	0.26 (0.25–0.27)	0.97 (0.81–1.16)	0.76	7.27 (7.21–7.33)	1.05 (0.96–1.16)	0.26
Mud	0.31 (0.28–0.33)	1	–	3.59 (3.51–3.66)	1	
Mix	0.21 (0.20–0.23)	0.93 (1.73–1.13)	0.45	4.70 (4.64–4.77)	0.96 (0.87–1.06)	0.44
Inhabitants						
(1–3)	0.25 (0.23–0.28)	1		6.30 (6.21–6.39)	1	
(4–6)	0.21 (0.20–0.22)	0.91 (0.78–1.07)	0.25	5.11 (5.07–5.16)	0.95 (0.88–1.02)	0.14
(7–10)	0.22 (0.21–0.23)	0.99 (0.84–1.16)	0.87	5.07 (5.03–5.12)	0.97 (0.90–1.05)	0.42
≥ 11	0.18 (0.16–0.20)	0.91 (0.74–1.12)	0.36	4.71 (4.63–4.79)	0.94 (0.85–1.03)	0.19
Hole on wall						
Yes	0.23 (0.22–0.24)	1.03 (0.93–1.14)	<0.0001	6.86 (6.81–6.92)	0.98 (0.92–1.03)	0.40
No	0.21 (0.20–0.22)	1	–	4.59 (4.56–4.62)	1	–
Eaves status						
Closed	0.21 (0.20–0.22)	1		4.45 (4.41–4.49)	1	
Opened	0.23 (0.22–0.23)	1.12 (1.02–1.24)	0.014	6.16 (6.12–6.20)	1.04 (0.99–1.09)	0.09
Ceiling status						
Yes	0.17 (0.16–0.18)	1		4.83 (4.78–4.87)	1	
No	0.25 (0.24–0.26)	1.37 (1.24–1.52)	<0.0001	5.88 (5.84–5.92)	1.06 (0.1–1.12)	0.05
Window status						
Screened	0.19 (0.17–0.20)	1		5.16 (5.09–5.22)	1	
Unscreened	0.22 (0.21–0.23)	1.13 (0.99–1.30)	0.66	5.40 (5.37–5.42)	1.06 (0.98–1.14)	0.15
Coverage of LLINs						
Yes	0.22 (0.21–0.22)	1		5.15 (5.12–5.18)	1	
No	0.20 (0.18–0.22)	0.91 (0.77–1.05)	0.20	6.05 (5.96–6.13)	0.98 (0.92–1.05)	0.62
Use of LLINs						
Yes	0.22 (0.21–0.22)	1		5.37 (5.34–5.40)	1	
No	0.20 (0.18–0.22)	1.01 (0.86–1.17)	0.94	4.35 (4.27–4.42)	0.99 (0.92–1.07)	0.79
Vegetation						
Yes	0.2 (0.20–0.21)	1.14 (1.01–1.28)	0.03	5.71 (5.67–5.74)	1.07 (1.01–1.13)	0.02
No	0.24 (0.22–0.25)	1	–	4.36 (4.31–4.42)	1	–
Breeding sites						
Yes	0.21 (0.21–0.22)	1.004(0.89–1.13)	0.95	5.63 (5.60–5.66)	1.06 (1.00–1.13)	0.04
No	0.23 (0.21–0.24)	1	–	4.51 (4.45–4.57)	–	–
Household heads’education level						
Illiterate	0.32 (0.28–0.36)	1		4.23 (4.11–4.35)	1	
Primary	0.20 (0.19–0.22)	0.88 (070–1.11)	0.29	5.56 (5.50–5.63)	1.09 (0.96–1.24)	0.17
Secondary	0.22 (0.21–0.22)	0.79 (0.64–0.98)	0.04	5.55 (5.50–5.59)	1.08 (0.95–1.22)	0.23
University	0.18 (0.16–0.19)	0.71 (0.56–0.92)	0.009	4.72 (4.65–4.79)	1.03 (0.90–1.18)	0.62
Bedroom						
< 5	0.22 (0.21–0.23)	1		5.29 (5.26–5.32)	1	
≥ 5	0.15 (0.13–0.17)	0.77 (0.65–0.94)	0.008	5.89 (5.80–5.99)	1.00 (0.93–1.09)	0.94
Window						
< 5	0.21 (0.20–0.21)	1		5.18 (5.15–5.21)	1	
≥ 5	0.22 (0.21–0.23)	0.96 (0.86–1.06)	0.4	5.73 (5.68–5.78)	0.99 (0.95–1.05)	0.96

*House characteristics and Culicines mosquitoes’ abundance* Culicines, particularly *Culex*, were found to be predominant in houses made with wood walls than in houses constructed with mud materials. Bivariate analysis indicated that the absence of ceiling (RR = 1.06, 95% CIs 0.1–1.12; P = 0.05), presence of vegetation (RR = 1.07, 95% CIs 1.01–1.13; P = 0.02) and breeding sites (RR = 1.06, 95% CIs 1.00–1.13; P = 0.04) close to houses were associated with Culicines presence in houses (Table 3). Multivariate analysis showed on their part that holes on the walls and the absence of screens on windows were the characteristics associated with the presence of Culicines in houses (P < 0.05).

*House characteristics and malaria parasites infection rate in Anophelines* Out of the 3557 Anophelines tested for *Plasmodium* infectivity using ELISA CSP, 80 were found infected; amongst which, 70 were *An. gambiae s.l.* and the remaining *An. funestus s.l..* The EIR varied from 0.005 to 0.011 infected bites/person/night (ib/p/n). When binary comparisons were conducted, the number of infected mosquitoes was not found to vary significantly according to the different house characteristics except in the case of comparison of houses with screened vs unscreened windows (P < 0.034). Table 4 presents the distribution of mosquitoes recorded infected according to house characteristics.

**Table 4:** Comparison of the distribution of mosquitoes detected infected according to house characteristics

House characteristics	Tested	Positive	Infection rate	EIR*	Odds ratio	(95% CI)	P value
Walls cement	1871	41	2.19	0.007	1	(0.45–2.25)	0.99
Mix	390	10	2.56	0.011	1.18	(0.44–3.13)	0.66
Mud	320	7	2.19	0.011	1	1	–
Wood	976	20	2.05	0.007	0.94	(0.39–223)	0.88
Occupants							
(1–3)	336	4	1.19	0.005	1		–
(4–6)	1650	35	2.12	0.007	1.78	(0.63–5.05)	0.27
(7–10)	1121	32	2.85	0.010	2.40	(0.84–6.83)	0.09
≥ 11	316	6	1.90	0.005	1.59	(0.45–5.70)	0.47
Hole on wall							
No	2195	51	2.32	0.008	1.03	(0.64–1.65)	0.90
Yes	1199	27	2.25	0.008	1		
Eaves status							
Closed	1200	31	2.58	0.009	1		
Opened	2271	48	2.11	0.008	0.82	(0.52–1.29)	0.39
Ceilings status							
No	2524	55	2.18	0.009	0.85	(0.53–1.37)	0.5
Yes	977	25	2.56	0.007	1		–
Windows status							
Screened	530	19	3.58	0.011	1		–
Unscreened	2906	59	2.03	0.007	0.57	(0.33–0.94)	0.03
Used of LLINs							
No	300	7	2.09	0.007	1.11	(0.51–2.45)	0.79
Yes	3008	63	2.33	0.007	1		–
Coverage of LLINs							
No	381	10	2.62	0.009	1.30	(0.66–2.57)	0.44
Yes	2979	60	2.01	0.007	1		–
Vegetation							
No	716	16	2.23	0.009	1.04	(0.59–1.82)	0.89
Yes	2702	58	2.15	0.007	1		–
Breeding sites							
No	600	11	1.83	0.007	0.84	(0.44–1.61)	0.60
Yes	2893	63	2.18	0.007	1		–
Household heads’education							
Illiterate	209	5	2.39	0.01	1		
Primary	656	12	1.83	0.006	0.76	(0.26–2.18)	0.61
Secondary	1621	36	2.22	0.008	0.93	(0.36–2.39)	0.87
University	389	13	3.34	0.01	1.41	(0.50–4.01)	0.52
Bedroom							
< 5	3070	66	2.15	0.008	1		
≥ 5	266	5	1.88	0.005	1.15	(0.35–2.18)	0.77
Window							
< 5	2301	50	2.17	0.007	1		
≥ 5	1025	23	2.24	0.008	0.97	(0.59–1.59)	0.9

Tested: number of mosquitoes screened, Positive: the number of mosquitoes found with *Plasmodium falciparum.* EIR*: Entomological Inoculation Rate: infected bites/person/night (ib/p/n)

## Discussion

House characteristics have often been reported to influence mosquito distribution [39–41]. Yet there are so far not enough studies assessing the influence of house characteristics on mosquito distribution in sub-Saharan Africa cities. In the city of Yaoundé several mosquito species are present all year round and are responsible for high mosquito burden. These include *Culex*, *Aedes* and Anophelines species [11, 42, 43]. Up to five Anophelines species were recorded during the study *An. gambiae, An. coluzzii, An. funestus, An. leesoni* and *An. ziemanni. Anopheles gambiae s.l.* was the most abundant group. The following was in accordance with studies conducted so far [11]. The densities of both Culicines and Anophelines were influenced by rainfall and was consistent with studies conducted so far in the city of Yaoundé [11, 42]. Despite the fact that more and more people stay outdoor very late in the night, high mosquito density was recorded indoor. The occurrence of mosquitoes inside houses was strongly associated with house characteristics and location. The risk of being bitten by mosquitoes was lower in houses constructed with cement walls or mix materials than in those constructed with mud or plank. The findings are consistent with the work of previous authors suggesting increase protection provided by house improvement [15]. Several parameters including presence of holes on the walls, the number of windows, the presence of opened eaves or breeding sites close to houses were all found associated with increase indoor mosquito abundance. In previous studies, parameters such as cooler environment, darkness, presence of crevices, house occupancy were also found to favor mosquitoes distribution and increased the risk of malaria transmission [44, 45]. One of the main factors responsible for high abundance of mosquitoes in houses was the number of people living per house [41, 46, 47]. In controlled experimental hut trials, it was demonstrated that houses with high occupancy tend to have more mosquitoes than those with low occupancy [22, 46]. In the present study, no similar association was recorded between house occupancy and density of mosquito per house this could have been confounded by the type of house since high densities of people were recorded in houses constructed with cement blocks which were well isolated compare to the other type of houses. The presence of screens on the window and the limited number of window in houses and close eaves were all found to increase protection from mosquito bites as reported elsewhere [48].

From the analysis, it also appeared that additional factors such as the number of bedrooms in houses and the education level of the household heads were also influencing the presence of both Anophelines and Culicines in houses. Households where the household head had the primary school level where found to be more exposed to high mosquito nuisance because they had a low socio-economic status and the majority had their houses constructed in lowland areas. Similar relationship between exposition to mosquito burden and the socio-economic status of the household have been reported in previous studies [49]. The number of bedrooms per house was found to be negatively correlated to the density of mosquito in houses and could result from the fact that in smaller houses the limited space available and the concentration of odours from people sleeping, could attract mosquitoes and also increase the efficiency of CDC light traps [41].

When comparing factors closely influencing the presence of Culicines and Anophelines in houses, it appeared that the absence of ceiling under the roof was one of the main factors affecting the abundance of host-seeking Anophelines in houses. For Culicines, the following factors were the predominant factors affecting their presence in houses: the type of house, the absence of screens on windows and the presence of holes on the walls. The following suggest different entry points for *Culex* and Anophelines species. Indeed *An. gambiae s.l.* have often been associated with specific entry points including open eaves [21] and absence of ceiling [48]. In Kenya, installation of ceiling with insecticide impregnated netting in sleeping room was found to reduce the indoor density of *An. gambiae s.l.* by about 76–82% [50]. Hence closed eaves, improved doors and ceiling could be efficient means to avoid Anophelines bites [50], especially in low malaria transmission settings where people do not use regularly LLINs due to low nuisance [51, 52]. The use of simple measures such as mud to seal all house gaps to limit entry points for mosquitoes could be promoted to fight against mosquito nuisance in traditional houses [26, 53]. Improving housing (through the replacement traditional building materials by modern material or construction of modern houses) could also be considered as an alternative vector management option to supplement current malaria control strategies in sub-Saharan Africa [39].

Possession of LLINs was found associated with reduce mosquito densities in houses. Treated nets have the capacity of killing and repelling mosquitoes and are recognized as a good mean for protecting against malaria transmission. Yet bed nets efficacy in Yaoundé could be affected by the rapid expansion of insecticide resistance in both *An. gambiae* and *Culex* species [42, 54].

The proportion of infected mosquitoes was not significantly different according to house characteristics. However, because of the high and frequent burden encountered by people living in poorly constructed houses they could be more exposed to malaria transmission risk than people living in modern constructions with limited nuisance. Yet because modern constructed houses are often situated next or surrounded by informal settlements or shanty towns, people living in high standard houses could be exposed as well to the same risk as people living in informal settlements. This stresses the need for more community efforts through the cleaning of the nearby environment to reduce the risk of malaria transmission. Providing a safe, reliable water supply system, and a better management of the nearby environment through the cleaning of drains, the elimination of domestic wastes or standing water collections around houses could be essential steps toward the fight against mosquito burden in urban settings [55].

A certain number of limitations could have affected the interpretation of the data. The fact that collections were done only indoor did not allowed comparison with outdoor mosquito densities. During the present study CDC light traps were used these traps are considered to be less efficient as compare to CDC UV traps and human landing catches [11, 56]. Also CDC light traps are known to be less efficient for collecting Anophelines compare to *Culex* this could have underestimated the density of Anophelines entering houses each night [57, 58]. The study also did not document the socioeconomic status of participants and the frequent use of repellent, coils or insecticide sprays by the population all this could have introduce some bias.

## Conclusion

The present study somewhat indicated that, poor housing in the city of Yaoundé, increase the risk of mosquito nuisance and disease transmission. Blocking entering points for mosquitoes such as closing eaves, placing a ceiling under the roof, putting screens on windows or constructing with cement walls could be essential improvements which could substantially reduce the density of mosquitoes entering houses. In the perspective of malaria elimination, promoting better housing should also be integrated in strategies to complement existing ones. So far there have not been many initiatives promoting improvement of houses for malaria and other vector borne diseases control in Cameroon. It becomes urgent that more initiatives been taken to encourage or sensitize the population on how they can improve their houses in order to reduce mosquito nuisance and prevent disease transmission. Future research should evaluate the protective effect of specific house features and incremental housing improvements associated with socio-economic development.

## Data Availability

Not applicable.

## Abbreviations

LLINs: Long-lasting insecticidal nets
CDC-LT: Centre for Diseases Control light traps
